# Unsupervised Monitoring Vegetation after the Closure of an Ore Processing Site with Multi-Temporal Optical Remote Sensing

**DOI:** 10.3390/s20174800

**Published:** 2020-08-25

**Authors:** Sophie Fabre, Rollin Gimenez, Arnaud Elger, Thomas Rivière

**Affiliations:** 1 Onera, Département Optique et Techniques Associées, 2 Avenue Edouard Belin, 31055 Toulouse, France; rollin.gimenez@onera.fr (R.G.); thomas.riviere@onera.fr (T.R.); 2 EcoLab, Université de Toulouse, CNRS, INPT, UPS, 31055 Toulouse, France; arnaud.elger@univ-tlse3.fr

**Keywords:** vegetation survey, change detection, fusion, former mining site, trace metal elements, multispectral, multitemporal, Sentinel-2 satellite

## Abstract

Ore processing is a source of soil heavy metal pollution. Vegetation traits (structural characteristics such as spatial cover and repartition; biochemical parameters—pigment and water contents, growth rate, phenological cycle…) and plant species identity are indirect and powerful indicators of residual contamination detection in soil. Multi-temporal multispectral satellite imagery, such as the Sentinel-2 time series, is an operational environment monitoring system widely used to access vegetation traits and ensure vegetation surveillance across large areas. For this purpose, methodology based on a multi-temporal fusion method at the feature level is applied to vegetation monitoring for several years from the closure and revegetation of an ore processing site. Features are defined by 26 spectral indices from the literature and seasonal and annual change detection maps are inferred. Three indices—CI_red-edge_ (CIREDEDGE), IRECI (Inverted Red-Edge Chlorophyll Index) and PSRI (Plant Senescence Reflectance Index)—are particularly suitable for detecting changes spatially and temporally across the study area. The analysis is conducted separately for phyto-stabilized vegetation zones and natural vegetation zones. Global and specific changes are emphasized and explained by information provided by the site operator or meteorological conditions.

## 1. Introduction

The state of vegetation, its survey and mapping are of growing interest to local territory stakeholders. Vegetation maps are an essential tool in the land use planning, creation and management of protected areas and in the monitoring of natural or polluted sites. Changes in vegetation provide information on the climatic, edaphic, geological and physiographic characteristics of an area [1]. In the natural environment, plants react to a multitude of biotic and abiotic factors.

The presence of pollutants in the growth medium (soil or water) can prove stressful for the vegetation and have direct physiological effects on plants (visual symptoms, photosynthetic process alteration, impact on development…). These effects result from the contact of the pollutants with the plants’ roots and their assimilation in the tissues [2,3,4]. Soil pollutants can also induce indirect effects on vegetation by changing soil properties such as the water regime and nitrogen availability to the plant [5]. Vegetation is then considered as an indicator of soil pollution which can be detected by means of optical imaging, with the changes in the characteristics of vegetation (pigment content, cell structure, water content, physiological state...) altering its optical properties [6,7,8,9,10,11,12].

Vegetation mapping and monitoring nearby mine sites are of interest in all phases of mining, from the planning (assessing biodiversity in the area, locating mining infrastructure, having a reference map beforehand to assess the environmental impact afterwards), mining and processing of ores (detecting potential contamination through their impact on vegetation) to closure and reclamation (providing information on the results of rehabilitation or detecting and controlling residual contaminants through vegetation health) [13,14]. In rural regions, mining activities are well documented as one of the most significant sources of soil heavy metal pollution [15,16,17].

Several works have reviewed the effects of trace metal elements, so-called heavy metals (HM), on vegetation reflectance [4,9]. The increase in HM in the soil has the effect of reducing the photosynthetic activity and chlorophyll concentration. Chlorophyll a and b, main pigments in leaves, present two light absorption peaks at 0.44–0.45 µm (blue) and 0.65–0.67 µm (red) [18]. The *red edge*, usually centered around 0.72 µm and identified by a fast-growing reflectance between 0.67 µm and 0.76µm, is employed through many approaches, based on spectral indices or radiative transfer models, to characterize leaf chlorophyll content variations [9,18,19]. Some experiments have also highlighted the interest of the Near InfraRed domain (NIR) plateau and the water absorption bands in the Short-Wave InfraRed domain (SWIR) to detect metal-stressed vegetation [20]. Vegetation sensitivity to HM depends on the contamination level, the species, the phase within the growth cycle and the environment [21].

Vegetation indices are simple or normalized reflectance ratios involving two or more spectral bands. Indices are applied on reflectance values or on transformations such as reflectance derivate or Continuum Removal (CR) [22]. They provide information on vegetation growth and stress due to their sensitivity to specific traits such as leaf chlorophyll or moisture contents, vegetation cover or biomass… Spectral vegetation indices have both spatial and temporal dimensions and can be used to identify changes in vegetation phenology [23]. Reflectance changes can then be related to the contaminants present in the growing environment through vegetation indices [9]. The most widely known and used index in optical remote sensing is the Normalized Difference Vegetation Index (NDVI) [24]. NDVI is an overall standardized way in which to measure healthy vegetation in a wide range of applications. Many other indices have been proposed to compensate for the disadvantages of NDVI related to its sensitivity to soil brightness and its saturation when used for dense canopy cover [25]. Some of these indices, such as Photochemical Reflectance Index (PRI) or Ratio Vegetation Index (RVI), have been applied on hyperspectral or multispectral reflectance data to detect vegetation stress related to heavy metals [9,26]. Recent indices have also been proposed for HM-stress detection in rice crops such as Heavy Metal Stress Sensitive Index (HMSSI), which is the ratio between two existing red-edge indices CI_red-edge_ (named CIREDEDGE) and PSRI ((Plant Senescence Reflectance Index) or Heavy metal Cd stress-sensitive Spectral Index (HCSI) [27,28]. It is difficult to select one or more indices from the large number existing in the literature, as the index performance depends on the context defined by the species, the pollutant, the pollution level and the environment.

Clustering analysis is an unsupervised method without the need for a priori information that is applied to vegetation monitoring and classification [29,30,31]. Pixels are grouped according to their similarities and a label is assigned to each group. These similarities concern spatial patterns, spectral signatures, texture and even temporal information [32]. The clustering algorithms largely used in remote sensing can be classified in three main classes: centroid-based methods such as K-means algorithm, neighborhood-based methods and hierarchical clustering [33,34,35,36]. The K-means approach is the best known clustering algorithm widely used in the last few decades [29,30,31,37], even if it can be unstable for large data sets. The two other classes limit this drawback but have higher computational costs. In order to enhance the performance and reduce processing time without losing variability, dimension reduction techniques such as Principal Component Analysis (PCA) or spectral indices are often applied before clustering [30,35,37]. Moreover, the exploitation of the vegetation spectral indices rather than spectral signatures allows the clustering algorithm to focus only on specific vegetation traits [33].

A high temporal resolution can provide an important advantage in capturing the dynamic information on vegetation states [38]. Time series of medium spatial resolution optical data (e.g., decametric spatial resolution) demonstrated a high capacity—for example, in providing inter-annual variations in the phenological stages from green-up to senescence [39], illustrating vegetation growth [40], or quantifying the temporal and spatial trends of vegetation cover [6,41].

Satellite-derived vegetation indices with a high temporal coverage provide an opportunity with which to obtain the biophysical parameters of vegetation over large areas (regional or global). The Sentinel-2 (S-2) (A and B) multispectral instruments, with a spatial resolution of 10 and 20 m depending on the spectral domains and a high temporal coverage (five days revisit time), are one of the best ways in which to detect inter-annual and intra-annual vegetation changes as well as stressed vegetation [7,33,42]. The presence of the red-edge bands provides the opportunity for HM stress monitoring [27]. The exploitation of multi-temporal data should make it possible to reduce false alarms that may be linked to other sources of stress, such as weather-related stress (flood, drought…) or pests and diseases, also known as abrupt stressors [28]. Abrupt stressors can affect vegetation over one or several growth cycles or one whole year [43]. Soil HM induces stable stress with a consistent long-term influence on vegetation growth.

Recent works introduce clustering to process multitemporal remote sensing and analysis land cover evolution, essentially for agricultural monitoring [31,37] and land use change [29,30]. Such time series data mining techniques allow us, by trend analysis, to examine shocks and unexpected variations or to detect anomalies and stable similar patterns. In agriculture, this information permits us to distinguish dissimilar levels of vegetation cover towards parcels as well as identify the planting pattern and phenology anomalies across development stages [29,31]. Times series are exploited by selecting sets of dates appropriate to the information to highlight [31,37]. The exploitation of multiple dates provides then the evolution between the selected periods.

Change detection is the process that analyses multi-temporal remote sensing images acquired over the same area in order to identify the changes occurring between the considered acquisitions dates [44]. Many publications show a comprehensive review of the change detection techniques [45,46,47,48]. These techniques are fusion methods in the time domain and can be classified according to the fusion level, whether feature-level or decision-level fusion [49]. Feature-level fusion methods are unsupervised automatic detection methods avoiding the need to perform in situ measurements and based on change indicators chosen to enhance multi-temporal information. Decision-level fusion methods are essentially made of supervised or semi/partially supervised/unsupervised classification methods. They include post-classification comparison, supervised direct multi-date classification and compound classification [46]. Supervised classification approaches improve performance in comparison to the unsupervised ones. They are, however, less usable in an operational context and depend on the learning base quality (number of samples, distribution of samples per class…).

Selecting an appropriate change detection technique depends on many factors: the study objective and the context, the size of the study area, the multi-temporal image spatial resolution, the in situ information availability, etc. In this work, change detection is applied in order to automatically identify the long-term changes related to behavior modifications between time series, using the temporal signature of land cover, and also to monitor seasonal/annual changes without a priori information about the change (localization, impact date, etc.). The change detection method based on feature-level fusion is suited to our needs. Several mathematical operators, relying mainly on the difference operator, are provided in the literature to define suitable change indicators [46]. Spectral indices have been used as detection indicators in order to highlight changes in many applications (land or forest cover change, burned area extraction...) [30,50,51,52]. The fusion of multi-temporal data can then be performed by image comparison [46]. In order to provide simple and fast detection maps, different operations (such as ratio, difference) can be applied to the original images or to the detection index images [45,47].

The main purpose of this study is to propose an unsupervised methodology applied on multispectral satellite time series to monitor vegetation for several years from the closure and revegetation of an ore processing site. This methodology implements, on the one hand, a simple and fast multi-temporal fusion method at the feature level (features inferred from spectral index selection) to detect simultaneously abrupt and longer-term changes in the vegetation development and traits (cover density, biochemical characteristics and phenology stages). On the other hand, a clustering recognition of spatial patterns within areas of the study site is provided for retrieving temporally stable (constant) patterns over the season and the year. The S-2 time series presents the geometric details and the spectral information required for jointly operating spectral, spatial and temporal information. The high temporal repeatability ensures both an analysis of the inter-annual variability of habitat changes over a large temporal period and an analysis of the habitat phenology over one year. Some areas of the study site have been phyto-stabilized, whilst in other areas natural (spontaneous) vegetation has developed. Phyto-stabilization has been enhanced by using soil amendments that immobilize HM combined with plant species that are tolerant of high levels of contaminants and low-fertility soils [13]. This phyto-management process allows us to partially confine HM by limiting its dissemination in the environment (through wind or water erosion, leaching).

The work is divided into several stages:

- Creation of the Sentinel-2 time series covering each season for many years;
- Construction of the spectral index base composed of numerous indices specified or used in various contexts (environment monitoring, biodiversity assessment, abiotic impact analysis, precision agriculture…);
- Development of an unsupervised diagnostic methodology based on the multi-temporal fusion methods for change detection and clustering recognition for pattern identification;
- Analysis of the intra-annual and inter-annual variabilities of the most pertinent spectral indices for the studied context in order to define the most appropriate phenological stage and to detect changes in vegetation over several years (temporal evolution comparison of phyto-stabilized vs. natural vegetation areas).

## 2. Materials and Methods

### 2.1. Study Area

**Figure 1:**
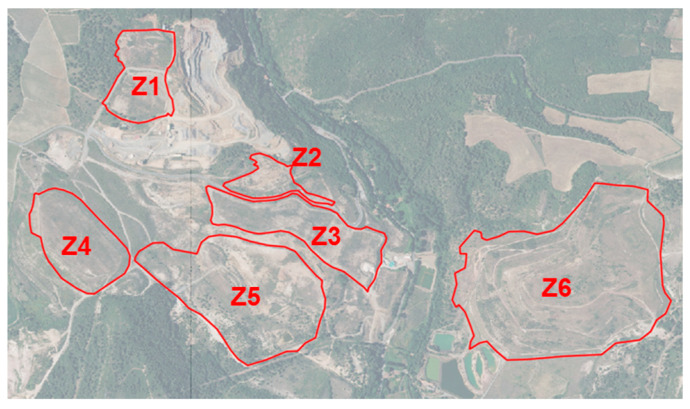
Former ore processing site and its six zones of interest, extracted from BD ORTHO^®^ 50 cm, ortho-photography provided by IGN (Institut Géographique National) with a spatial resolution of 50 cm—scale: 1:10,000. Area of each zone (in ha): Z1 (6.5), Z2 (2.8), Z3 (9.4), Z4 (8.5), Z5 (19.4), Z6 (35.3).

The study area is a former ore processing site in France that was closed in 2004 and is managed by a French public institute. In 2006, some areas of the site were phyto-stabilized. The site covers approximately 120 hectares. Six zones of interest defined by the institute were analyzed in this study (Figure 1). Z1, Z2 and Z5 are the three aided phyto-stabilized zones. The phyto-stabilization is realized at the same period over most of the areas. Some differences in the management mode concern zones Z1 and Z5. On the central part of Z1, natural uncontaminated soil was introduced. In Z5, only the northeastern half was phyto-stabilized by a steel shot mixture. Spontaneous vegetation developed on the other part of the site and, in addition, trees were planted in alignment. According to the maps provided by the institute, Z2 and Z5 have the highest metal signatures in their soil. In situ measurements cannot be provided because of the site sensitivity and for intellectual property reasons, but a map with two metal signature classes is provided in the result analysis. Z3, Z4 and Z6, identified as natural vegetation areas, have been vegetated with a mixture of local seeds on clean soil brought and the natural vegetation has developed over the years. Species are known for zones 2, 3, 5 and 6 and are provided afterwards.

In order to provide information on the relief of the study site, the BD-ALTI^®^ 25 m, Digital Terrain Model (DTM) provided by Institut Géographique National (IGN) and describing the relief of the French territory at a spatial resolution of 25 m, is processed by Geospatial Data Abstraction Library (GDAL) in order to extract the contour lines at 10 m elevation intervals [44]. The elevation in the study area ranges from approximately 180 to 280 m. Z6 has the greatest difference in height (about 75 m), and Z1 and Z4 present the lowest differences in height (about 15 m).

### 2.2. Sentinel-2 Satellite Imagery

The Sentinel-2A images covering the (0.4–2.5 µm) domain available on the study site are selected according to their quality (clear sky, processing level 2A surface reflectance products after geometric and atmospheric corrections) between May 2016 and May 2020—i.e., starting and ending in spring with, in theory, maximal green cover [53,54].

Some other considerations are taken into account when choosing the processed dates. For each year, the seasons must be the same in order to detect abrupt changes. Seasons must be properly sampled over the year to monitor the phenology evolutions. The choice is then made to select one image per season. The time series consists of 17 images listed in Table 1. No image is selected in February 2016 due to the presence of clouds.

**Figure 2:**
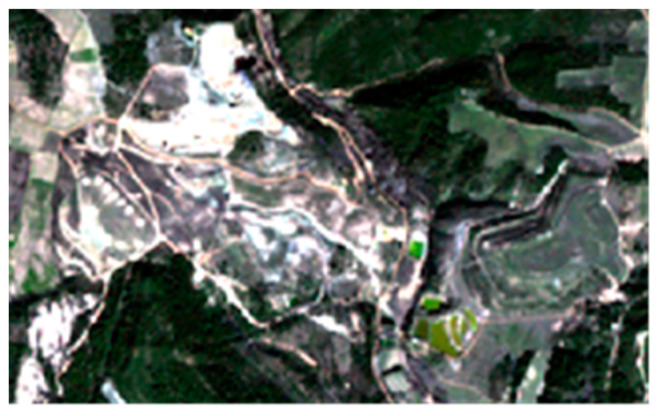
RGB (Red-Green-Blue) representation of a thumbnail image extracted from a processed Sentinel-2 image (10 m spatial resolution).

Ref. [7,55] provide the central wavelength positions and spatial resolutions for the Sentinel-2A spectral bands. A multispectral data cube (x, y positions and wavelengths) with a spatial resolution of 10 m is produced for each image referenced in Table 1. For this purpose, the spectral bands at 20 m spatial resolution are oversampled to a 10 m spatial resolution using a nearest neighborhood filter in order to preserve the spectral information [56]. For each image, a thumbnail image associated with the study site is extracted (Figure 2).

### 2.3. Methodology

As mentioned above, the most suitable method for our application case is a multi-temporal fusion method at the feature level. The fusion is processed in two stages:

1. Feature extraction: various spectral indices are used to highlight features;
2. Feature comparison: a mathematical operator is applied to highlight the changes that occurred in bi-temporal image pairs.

**Figure 3:**
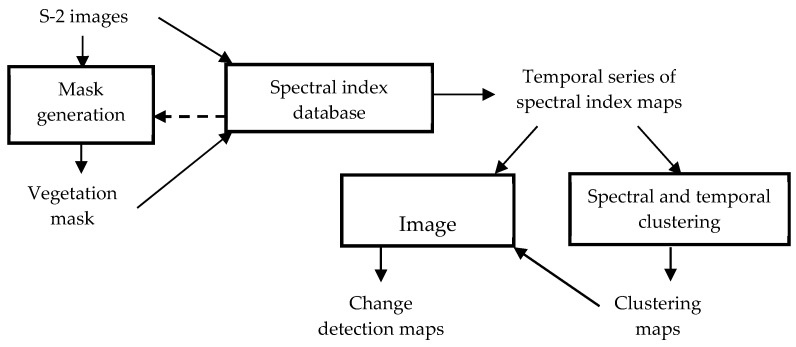
Methodology description.

The processing chain includes four modules (Figure 3): a mask generation module to identify the vegetation pixels to be processed thereafter, a spectral indices database to produce feature maps, a spectral and temporal clustering module and an image comparison module to detect change. The inputs are spectral reflectance images and the outputs are change detection maps.

#### 2.3.1. Mask Generation

For each S-2 image, a mask is generated so as to identify the vegetation pixels to be processed and to mask the pixels associated with other land use classes (bare soil, water, buildings…). The following combination of spectral indices is applied on the reflectance image to produce its corresponding mask:

- Normalized Built-up Area Index (NBAI) and Band Ratio for Built-up Area (BRBA) to identify road or building pixels [57,58];
- Normalized Difference Water Index (NDWI) and modified Normalized Difference Water Index (mNDWI) to highlight water areas [59];
- Soil Adjusted Vegetation Index (SAVI) and Dry Bareness Spectral Index (DBSI) to identify bare soil pixels [60,61];
- NDVI to provide vegetation cover pixels [24].

The combination of NDVI with indices related to other land use classes allows a reduction in the false alarm rate. A binary thresholding is applied to each index map, and a histogram is used to determine the appropriate threshold value. The index maps after thresholding are combined by an AND logical operator.

**Figure 4:**
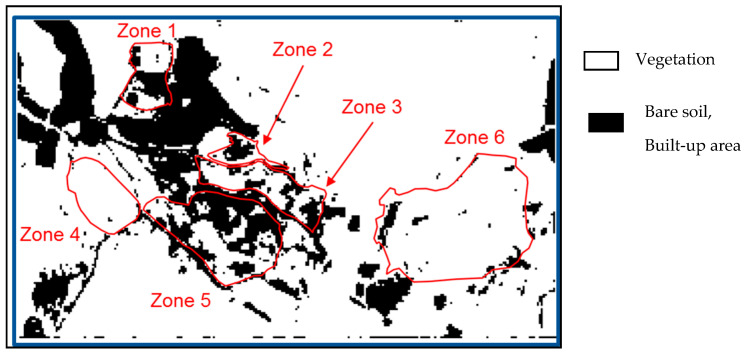
Vegetation mask from the multispectral image acquired on 21 May 2016.

Figure 4 shows the mask obtained from the multispectral image acquired on 21/05/2016. The black pixels represent non-vegetated areas and the vegetation pixels are white. The vegetation masks are analyzed and compared in the next to monitor the development and evolution of the spatial distribution of vegetation between 2016 and 2020.

#### 2.3.2. Spectral Index Database

Table 2 provides the description of the spectral indices database. Each index formulation is provided and the corresponding reference is cited. The S-2 spectral bands used by each index are provided ([7,55] defining the central wavelength and the spatial resolution for each band).

A total of 25 indices use spectral bands in the Visible-Near InfraRed (VNIR) spectral domain, and only one index, NBR, exploits the band B12 in the Short Wave InfraRed (SWIR) domain. There are many indices exploiting the red edge. This database includes:

- Indices specified for global vegetation monitoring such as NDVI [77] or MTCI (MERIS Terrestrial Chlorophyll Index) [74];
- Indices defined to characterize the impact of contaminants (heavy metals, hydrocarbons...) on vegetation such as EMEN2 (Red and green ratio) [66] or HSSMI (Heavy Metal Stress Sensitive Index) [27];
- Indices defined to estimate biochemical parameters (chlorophyll, anthocyanin…) such as ARVI [62] or SIPI [79].
- NDVI, widely used in many applications [24], is chosen as the reference index and the other indices are compared to this reference index. The best-performing indices are selected and the complementarity of the selected indices are analyzed.

#### 2.3.3. Spectral and Temporal Clustering

##### Description of the Clustering Methods

Clustering is used to regroup and classify pixels with the same spectral and temporal behaviors [29,30,31,80]. After applying a vegetation spectral index on the time series, for each pixel a matrix regrouping spectral and temporal information is constructed:

(1) $$M_{i,j}=\left[I_{i,j,t_{1}},\ldots .\;I_{i,j,t_{n}}\right],$$

where *I* is the considered spectral index or vector of several spectral indices, *i*,*j* are the coordinates of the pixel of interest and $t_{k}$ represents the date *k* of the time series composed of *n* dates k∈[1,n].

Several clustering methods used in the literature are compared in order to select the most adapted for our study (methods are provided by the scikit-learn library [81]). These methods are partitioned in three classes:

- Centroid-based methods, like the K-means algorithm [33,34]: This consists of randomly initializing K centroids to assign each data point (in this case $M_{i,j}$) to its nearest centroid according to the Euclidean distance. Then, the centroids are recomputed as the mean (in the case of the K-means algorithm) of the data point of each group. These steps are finally repeated until attempting a stopping criterion. For its efficiency, K-means is selected and its instability is solved by running 100 times (value empirically fixed) the algorithm with different centroid seeds and selecting the best output in terms of inertia [33]. Still in this class, another chosen method consists of adding a principal component analysis (PCA) algorithm to reduce the dimension of the time series without losing its variance before applying K-means;
- Neighborhood-based methods, such as spectral clustering [35,36]: The dataset is processed by creating a similarity graph by k-nearest neighbors (graph constructed by connecting each point to its K-nearest neighbors) or ε-neighborhood (graph obtained by associating to each point all points falling inside a circle of radius ε, where ε is a real value). Then, a corresponding Laplacian matrix is created to project the data onto a lower dimensional space, and K-means is applied on its rows. Both the ε-neighborhood and k-nearest neighbor methods are tested. The high computational cost obligates this class of method to provide for some adjustments such as image preprocessing [37,82]. In our case, the preprocessing consists of applying a spectral index.
- Hierarchical clustering algorithms: partitions are obtained by the successive groupings of objects (bottom-up approach such as agglomerative clustering, the algorithm chosen in our study) or by successive division (top-down approach) [33,34].

##### Performance Comparison of the Clustering Methods

**Figure 5:**
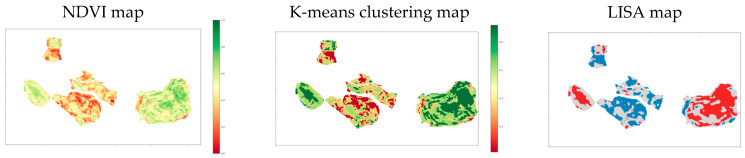
Comparison of the K-means clustering map with the LISA (Local indicators of spatial association) and NDVI (Normalized Difference Vegetation Index) maps. On LISA map: red clusters are pixels of high NDVI values (in comparison with the average NDVI value) surrounded by pixels of high NDVI values, orange cluster are pixels of high values surrounded by pixels of low values, pale blue those of low values surrounded by pixels of high values and blue those of low values in a group of pixel of blue values.

According to former works on clustering, evaluating the accuracy of a clustering classification is far from easy because clustering results do not necessary represent in situ measurable information [37]. Thus, to select an algorithm and fix its input parameters, three criteria defined according to published studies are considered [33,37]:

- The computational cost of each algorithm.
- The internal quality of clusters, defined by analyzing the compactness of each cluster and the separation between clusters. This information is evaluated by the silhouette score (or index), which indicates if a pixel belongs to the right cluster [83]. A score close to 1 indicates a good clustering. The average of these scores over all the data indicates if the method has appropriately clustered the data.
- The external quality of clusters, defined by comparing the clusters to externally supplied information. To do this, the Moran’s Index (Moran’s I) is applied to spectral index maps. It is a measure of spatial autocorrelation which can be calculated over all the data (global Moran’s I) or in a specific zone (local Moran’s I or LISA Local Indicators of Spatial Association) [84]. While the global index gives the spatial correlation of the entire dataset by returning a number between -1 and 1, the local index allows drawing a correlation map. Both are used to measure the efficiency of our clustering. The LISA map returns a map described by five classes (Figure 5). Two, usually called High-High or Low-Low, indicate regions which contribute significantly to a positive global spatial autocorrelation outcome; two others, called High-Low and Low-High, indicate those which contribute significantly to a negative autocorrelation outcome and one that indicate those with an insignificant contribution. Thus, the comparison of the classes obtained by the Moran index with the clusters allows us to verify the relevance of our clusters.

This analysis is performed for a single date or combined dates and spectral indices in order to select the clustering algorithm among the following methods: K-means, PCA and K-means, spectral clustering with k-nearest neighbors, spectral clustering with ε-neighborhood, hierarchical clustering.

An example of the K-means performance evaluation is illustrated by Figure 5 for a specific date (21 May 2016). The silhouette score is equal to 0.53, which is a significant score. The global Moran’s I is equal to 0.87, which indicates strongly grouped and correlated NDVI values across the map. The LISA map shows that the clusters returned by the LISA method are almost the same as those returned by the K-means algorithm.

##### Clustering Method Selection

The simple K-means and principal component analysis followed by K-means are slightly quicker than the other methods. The silhouette scores are globally coherent according to the algorithms, but simple K-means presents barely better results than other algorithms. Finally, by comparing the maps returned by the different algorithms, all are consistent with Moran’s I. Small differences observed only are the boundaries between clusters. Since the simple K-means algorithm is slightly better in terms of computational time and silhouette score, this algorithm is selected.

##### Input Parameter

The cluster number, the input parameter of the K-means algorithm, has to be adapted to the application case. The silhouette score is initially used to fix the optimal cluster number. Two, three, four and five clusters are tested. The difference values of the silhouette score are not sufficient to decide the number of clusters on this criterion.

The cluster number selected has to represent the fluctuation range of the index values according to the season and the studied zones. Two or three clusters are not sufficient to represent the seasonal and inter-zones variabilities. Five clusters do not give more information than four clusters. Moreover, according to the literature [85], the NDVI value range is often divided into four classes: lower than 0.2 for bare soil, approximately 0.2 to 0.4 for sparse vegetation (like shrubs, grassland, or senescing crops), approximately between 0.4 and 0.6 for moderate vegetation, and approximately higher than 0.6 for a high density of green vegetation, such as that found in temperate and tropical forests or crops at their peak growth stage. This leads to us retaining four clusters.

#### 2.3.4. Image Comparison for Change Identification

Simple change detection techniques providing easy to interpret results are chosen [45]. A hypothesis of precise geometric registration between multi-temporal images to avoid false change areas being detected due to an image displacement is retained [47]. This assumption is realistic, as the multi-temporal images are acquired by a single instrument. The suggested change detection methods are based on the absolute, relative and normalized differences between two spectral index maps at two different dates. The difference is applied pixel by pixel. These methods are compared, and finally only the normalized difference is studied as normalization allows one to compare the difference values from one date to another. It is based on the following formulation:

(2) $$dif\_norm_{d1,d2}=\frac{I_{d1}-I_{d2}}{I_{d1}+I_{d2}}$$

where *d*_1_ and *d*_2_ are two different dates, and $I_{di}$ is the map associated with a given spectral index (Section 2.3.2) and the date *d_i_* (*i* = 1, 2). $I_{di}$ can represent the spectral and temporal clustering map (Section 2.3.3) associated with a given time series *d_i_.*

The vegetation masks associated with each date are combined by an OR operator in order to provide the vegetation mask of the detection map.

**Figure 6:**
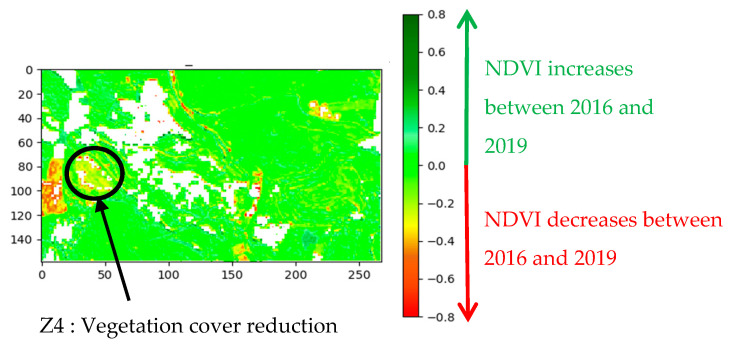
Example of detection map displaying the evolution of NDVI between spring 2016 and spring 2019 (reference year: 2016).

Figure 6 illustrates an example of change detection map obtained by applying the normalized difference to two NDVI maps. The decrease in the NDVI values in Z4 is quickly identified on the detection map.

#### 2.3.5. Data Analysis

A large volume of data has to be compared. A data analysis strategy is then put in place. Two types of result analysis are carried out:

- A global visual analysis and a visual analysis per zone of the various generated maps. The maps are vegetation masks (Section 2.3.1), spectral index maps (Section 2.3.2), change detection maps applied on spectral indices (Section 2.3.4), clustering maps (Section 2.3.3) and change detection applied on clustering maps (Section 2.3.4).
- A statistical analysis per zone. On one hand, the following statistics are calculated from the generated maps: mean, standard deviation (noticed STD), coefficient of variation (also known as RSD, Relative Standard Deviation) [86], median, first and third quartiles (Q1 and Q3, respectively). On the other hand, the temporal evolution of the median and mean are analyzed and median results are provided.

The statistics and maps obtained for the indices described in Table 2 are compared. At first, the indices characterized by temporal and spatial variabilities are chosen by a visual analysis of the index maps. Among these indices, the ones with similar temporal changes over seasons are grouped together by a statistical analysis of the index temporal evolution. For each group, the indices are compared in order to eliminate the indices leading to redundant information. Finally, the change detection maps and associated statistics obtained for the selected indices are then compared in order to define the best-suited indices for change detection.

Spectral and temporal clustering is applied to the selected indices (unique index or combination of indices) and a temporal series in order to highlight stable spatial patterns, monitor the annual evolution, and emphasize phenological dissimilarities over the year. Change detection is then applied to the clustering maps.

## 3. Results

Because of the significant amount of results obtained, only the fundamental outcomes are provided within this section. Results are completed by the Appendix A.

### 3.1. Temporal Evolution of the Vegetation Cover

Vegetation masks are used to monitor the temporal changes in the vegetation cover density and spatial distribution in each area.

**Figure 7:**
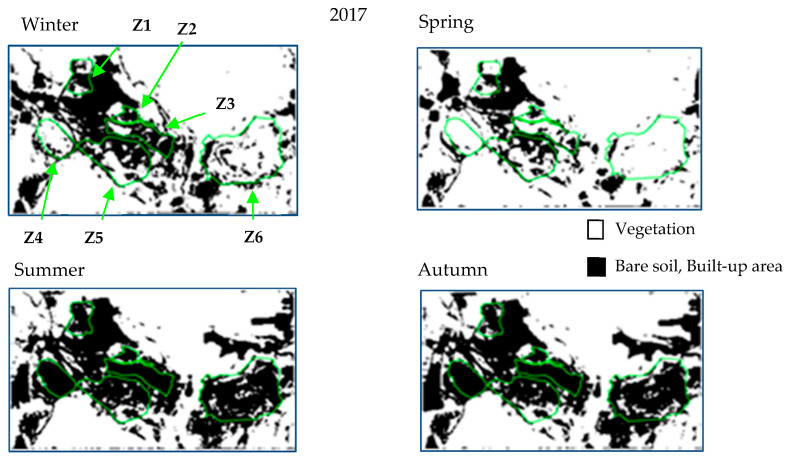
Seasonal evolution of the vegetation mask for the year 2017.

Figure 7 represents the seasonal evolution of vegetation for the year 2017. In summer and autumn, most parts of the monitored areas have no vegetation. The two seasons with the most significant vegetation cover are winter and spring. This observation is valid for each year. For this studied site, these seasons are those to favor for vegetation monitoring.

**Figure 8:**
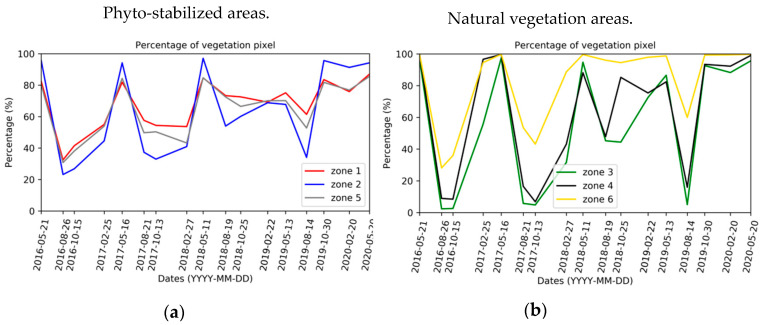
Temporal evolution of the percentage of vegetation pixels for each zone from 2016 to 2020, for phyto-stabilized (**a**) and natural vegetation (**b**) areas.

Figure 8 shows the temporal evolution of the percentage of vegetation pixels for each area. The same vegetation cycle is observed for phyto-stabilized and natural vegetation zones. Globally, this cycle corresponds to the reduction in vegetation cover in summer and growth in spring. Globally, there is an increase in vegetation cover over the years.

Larger deviations are identified between summer and spring for two zones with natural vegetation (zones 3 and 4). The phyto-stabilized zones and zone 6 with natural vegetation maintain a greater vegetation cover in summer and autumn than zones 3 and 4. Zone 6 contains well-developed trees, mainly pines and poplars, and Spanish brooms (*Spartiumjunceum)*. This explains the vegetation cover higher than 30% in summer and autumn. The difference in vegetation covers between zones 3 and 6 is explained by the presence of distinct species. The five dominant species in zone 3 are species present in the Mediterranean region: *Aphyllanthesmonspeliensis* (plant abundantly flowering in spring, forming rush-like clumps with blue flowers), *Bituminiariabituminosa* (a perennial plant that grows on the edges of fields or paths), *Dittrichiaviscosa* (an invasive perennial which flowers in late summer and early autumn, often growing in wasteland and rubble and along roadsides, forming abundant green tufts with yellow flower heads), *Pallenisspinosa* (an annual or biennial plant generally not more than 70 cm high with dark, stiff, straight stems with spaced hairs) and *Plantagolanceolata* (commonly known as Ribwort plantain, a perennial herbaceous plant of average size 15–50 cm, which exhibits variable forms depending on soil nutrient richness, sunlight exposure and soil moisture). Zone 3 also includes Spanish brooms and some developing trees (pines, poplars) on the edge of the zone.

The percentages of vegetation pixels in zone 1 are close to those in zone 5. Zones 1 and 5 have more vegetation pixels in summer–autumn than zone 2 and, conversely, fewer vegetation pixels in spring compared to zone 2 (differences in the order of 10% to 20% depending on the year considered until autumn 2019). After autumn 2019, the vegetation cover of zone 2 exceeds that of the other two areas. Zone 1 species have not been provided. Zones 2 and 5 are composed of the same species. The only dominant species common with those in zone 3 is *Dittrichiaviscosa*. The other three dominant species identified in zones 2 and 5 are *Blackstoniaperfoliata* (annual herbaceous plant that flowers from May to September with yellow flowers), *Securigeravaria* (perennial herbaceous plant that flowers from June to September), *Trisetumflavsecens* (perennial herbaceous plant of the *Poaceae* family, growing in thick clumps, up to 60 to 80 cm high). The presence of herbaceous vegetation in these areas explains the high pixel rates of vegetation cover in summer and autumn in comparison to the rates of zone 3.

**Figure 9:**
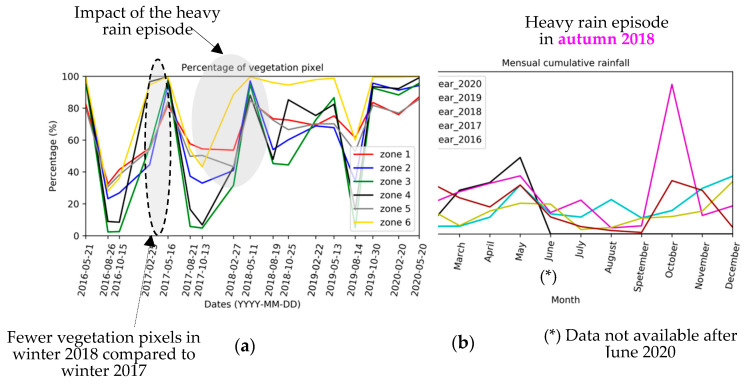
(**a**) Temporal evolution of the percentage of vegetation pixels by zone (between May 2016 and May 2020); (**b**) seasonal evolution of the monthly rainfall during 5 consecutive years (in mm).

After autumn 2018, an abrupt change in the vegetation cycle is observed. To find out the reason for this phenology alteration, the available meteorological data are analyzed. The change in vegetation cycle after autumn 2018 is explained by the heavy precipitations in October 2018, almost three times higher than the precipitations in October 2016 (Figure 9). Vegetation cover appears to increase overall across the years (Table 3). This increase is mostly highlighted after the autumn 2018 and can be correlated with the significant precipitation levels of autumn 2019 and spring 2020. A change in the phenological stages is observed and vegetation cover is increasing in autumn from 2018 (Table 3). Prior to this date, the vegetation cover in autumn was comparable to the one during summer. In spring, the vegetation cover is the most important and remains relatively stable over the years (maximum increase of 5%). There are low apparent differences between the phyto-stabilized and natural vegetation areas, except for the highest rates of increase in autumn and the lowest increases in winter and spring over these 4 years for the natural vegetation areas.

The evolution of the vegetation cover is analyzed as a function of the average monthly temperature. February 2018 is the coldest month within the analyzed period, and the increase in the number of vegetation pixels between autumn and winter 2018 is smaller than the increase observed in 2017 (Figure 9).

**Figure 10:**
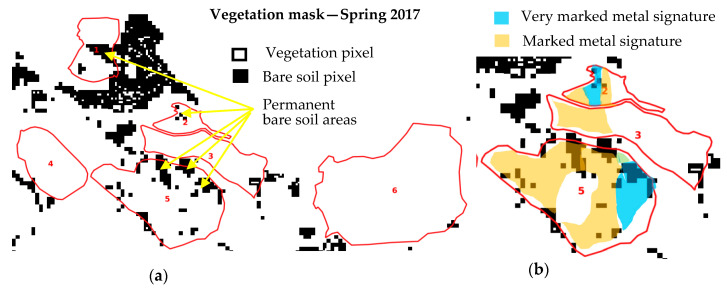
(**a**) Identification of areas without vegetation cover; (**b**) superposition of the metal signatures classes provided by in situ measurements.

An analysis of the vegetation masks reveals areas of permanent bare soil with no vegetation cover regardless of the year and the season. These areas are identified on Figure 10a and are related to the HM field measurements in zones 2, 3 and 5 (Figure 10b). The bare soil of zone 5 corresponds to marked metal signatures in soil. The areas with the highest HM contents are not necessarily identified as areas of permanent bare soil in the image time series. Some vegetation has the capacity to grow on HM-contaminated soil.

### 3.2. Spectral Index Selection

**Figure 11:**
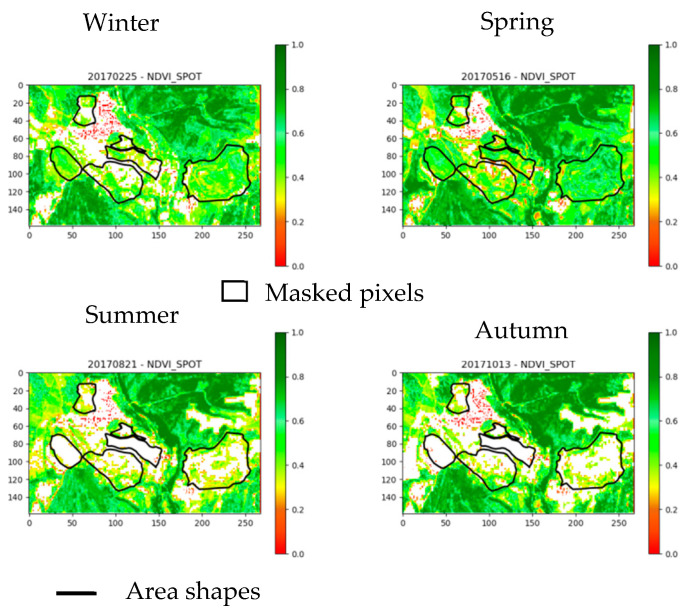
Seasonal evolution of NDVI reference index maps for the year 2017 (masked pixels corresponding to non-vegetation pixels, area shapes representing the contours of the study zones)**.**

The selection and evaluation of the spectral indices is carried out in several steps. Firstly, the NDVI reference index maps are visually analyzed. Figure 10 shows, for example, the seasonal evolution of the NDVI maps for the year 2017. In addition, the NDVI statistics (mean, standard deviation, RSD) are computed by the area and vegetation type (i.e., vegetation introduced for phyto-stabilisation, natural vegetation) (Section 2.3.4). Figure 11 provides the annual temporal evolution of NDVI statistics by zone.

The NDVI analysis leads to the following common observations throughout the study area:

- The NDVI index presents the same phenology stages for all zones except zone 5. NDVI increases in spring and decreases in summer.
- A change is observed in winter 2019; the increase in the NDVI value is less marked than those of the other years (a decrease is even observed for zones 4 and 5). This can be explained by the heavy rainfalls in autumn 2018. This abrupt change is also identified during the analysis of the percentage of vegetation pixels (Figure 11).
- Starting in autumn 2019, the NDVI increases compared to the other years (especially for zone 6), and this can be related to the regular rainfall over the period November 2019 to May 2020.
- The highest interquartile range (IQR) values are observed for dates when the percentage of vegetation pixels per zone is less than 20%.

**Figure 12:**
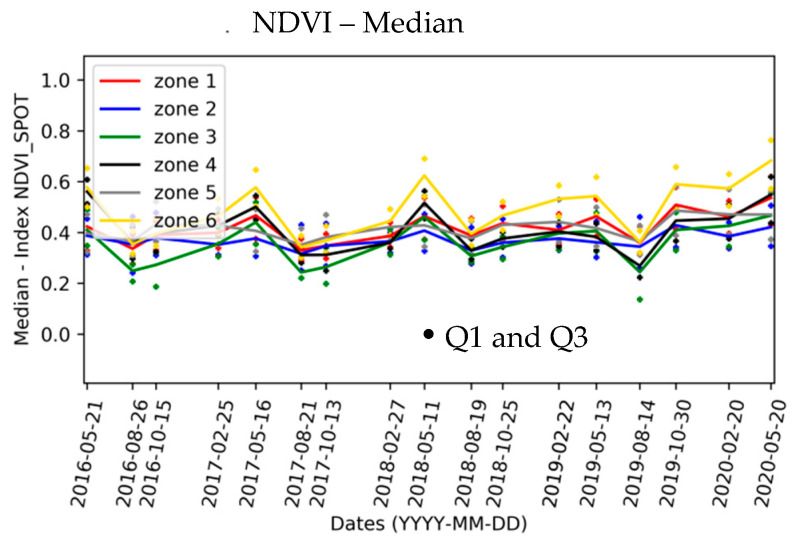
Multi-annual evolution of NDVI statistics (median, Q1, Q3) by zone.

For natural vegetation zones (zones 3, 4 and 6), the NDVI is generally higher than for the phyto-stabilized zones, except for zone 3. Zone 3 has the lowest NDVI values until autumn 2017. Zone 6 has the highest NDVI values from autumn 2016 and the highest numbers of vegetation pixels in summer and autumn (Figure 12). Zone 6 is the zone with the most developed and tallest vegetation.

For phyto-stabilized zones (zones 1, 2 and 5), the cycles are less pronounced and the differences in NDVI from one season to the next are smaller. There are areas for which the number of vegetation pixels remains greater than 20% whatever the season (Figure 9). Zone 5 has a different phenology compared to the other two zones. NDVI is high in autumn or winter (depending on the year) and decreases in spring. The in situ information indicates that zone 5 is the area with the highest species diversity.

**Figure 13:**
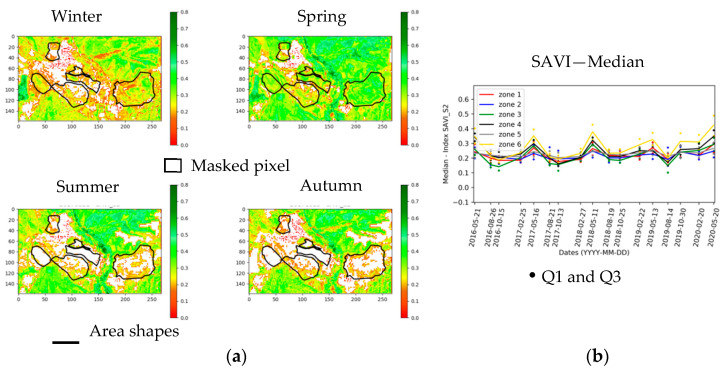
(**a**) Seasonal evolution of SAVI (Soil Adjusted Vegetation Index) for the year 2017; (**b**) multi-annual evolution of SAVI statistics by zone: median, Q1 and Q3.

In a second step, only indices with both spatial and temporal variabilities are chosen. For example, the SAVI index is selected (Figure 13a). The seasonal evolution of SAVI is different from that of NDVI owing to their formulations: in autumn SAVI decreases while NDVI increases (Figure 12 and Figure 13b). The lowest SAVI values are then obtained in autumn, whereas those of NDVI are obtained in summer. NDVI allows the assessment of biomass importance and the monitoring of chlorophyll activity. It is often used for moderately dense canopies (no apparent soil but not too dense to avoid a saturation effect), without the mixing of standing dry matter with green matter. SAVI introduces an adjustment factor to minimize the ground effect. When this factor is equal to 0, SAVI and NDVI formulations are the same. SAVI is sensitive to soil color and brightness and is adapted in the case of a low chlorophyll vegetation cover [62,71,87]. NDVI and SAVI are then separated into two groups. This leads to the grouping of the indices with similar temporal evolution as follows:

- Group 1: NDVI (reference index), GREEN_NDVI, ARVI, CIREDEGE, MTCI, NDRE;
- Group 2: SAVI, MSAVI, IRECI, MCARI_NEW;
- Group 3: VII;
- Group 4: PSRI.

The SWIR index is not kept as the difference in spatial resolutions between VNIR and SWIR [7,54] should contribute to its disqualification. HSSMI (Table 2), specified for HM detection from Sentinel-2 images, is not used because of the very high IQR and RSD values (RSD between 50% and 100%).

These groups of indices are analyzed in detail below. Every index of group 1 is compared with the reference index belonging to this group. For Group 2, the SAVI index is compared to the reference index in order to check the complementarity of this index group. The other indices of Group 2 are then compared to SAVI. For groups 3 and 4 consisting of a single index, they are compared with the reference index.

**Figure A1:**
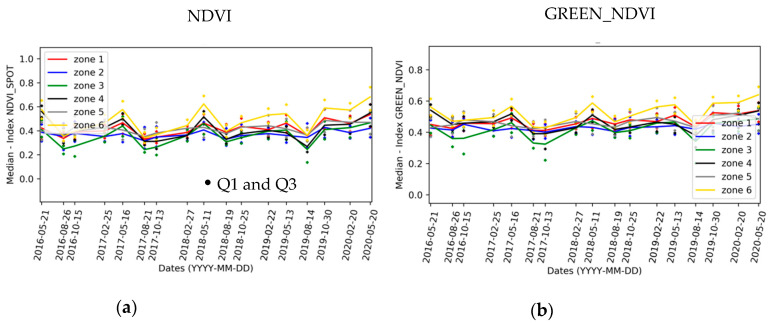
Multi-annual evolution of statistics (median, Q1, Q3) by zone: (**a**) NDVI; (**b**) GREEN_NDVI. The variation range of GREEN_NDVI is lower in comparison to NDVI’s one, which leads to a lower spatial variability, and the temporal differences are less pronounced.

Group 1 indices are compared to NDVI. Maps are visually compared as well as statistics. ARVI cannot be retained because its RSD values are twice as high as those of NDVI. The variation range of GREEN_NDVI is weaker than the one of NDVI, which leads to lower spatial variability and the temporal differences are less pronounced (Figure A1). GREEN_NDVI will not perform as well as NDVI in the detection of change. NDRE and NDVI provide the same information and the reference index is retained. CIREDEDGE presents a higher variation range, which highlights the temporal differences. This index, used to estimate chlorophyll, is retained in order to complete NDVI. MTCI supplies consistent information with CIREDEGE but provides higher RSD values. This analysis leads to the selection of CIREDEDGE in order to complete NDVI.

SAVI of Group 2 is compared to NDVI. Figure 4 compares the statistical values. In spring 2019 and the following seasons (exception of summer), for all zones, SAVI increases whilst NDVI varies differently depending on the considered zone. Zone 5 shows a temporal evolution of SAVI consistent with the zone 2 evolution, whereas the evolution of NDVI for zone 5 is not comparable to any other phyto-stabilized zone. The SAVI index is therefore complementary to the NDVI index.

The other indices of Group 2 are compared with SAVI. MSAVI is not retained because it provides the same information; the initial formulation of the SAVI index family is retained. The variation range of MCARI_NEW is lower and its RSD values are almost 1.7 times higher than those of NDVI. IRECI is of interest and is used to complete SAVI.

**Figure 14:**
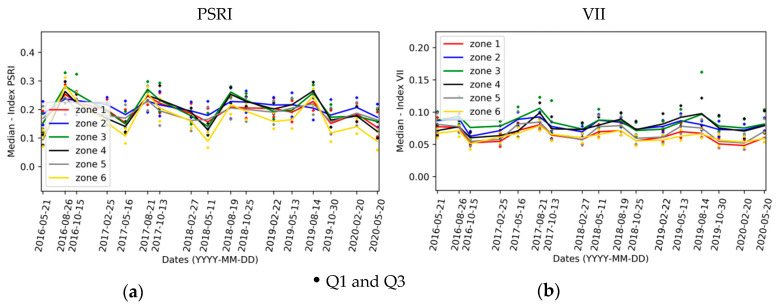
Multi-annual evolution of median indices by zone: (**a**) PSRI; (**b**) VII (Vegetation Inferiority Index).

Group 3 consists of a single index PSRI specified for senescent vegetation. PSRI is sensitive to the ratio between carotenes and chlorophylls. It shows an inverse seasonal evolution in comparison to NDVI (Figure 12 and Figure 14a): the lowest values are obtained in spring (in summer for NDVI) and the highest values in summer (in spring for NDVI). In addition, the areas with high NDVI values are the areas with low PSRI values (e.g., zone 6), which is explained by the role of each index. For zone 2, an increase in PSRI is observed for the date 2017/02/25, when the number of vegetation pixels is low. This difference is less pronounced when NDVI values are analyzed. The ranges variations of the IRQ and RSD values for PSRI and NDVI are similar. PSRI is complementary to NDVI.

Group 4 consists of a single VII index developed for monitoring the state of vegetation growth in a mining area using hyperspectral data [80]. VII has been adapted because in the original formulation, it uses the green bands from 497 to 635 nm and the NIR and SWIR bands from 700 to 1200 nm. In our case, according to the 60 m spatial resolution of the two spectral bands B9 and B10 (Table 2), the second spectral band covers the domain from 704 nm to 865 nm. The VII seasonal evolution is different from the NDVI evolution. In autumn 2017 and for the following dates, zones 2, 3 and 4 present equivalent VII levels and global variations; the VII temporal evolution is comparable for zones 1, 5 and 6.

The high VII standard deviation values correspond to areas and dates with few pixels of vegetation. RSD values assessed from VII are lower for phyto-stabilized areas than those calculated from NDVI and, conversely, for areas with natural vegetation. This index is complementary to NDVI.

To conclude, the retained indices by group, each group corresponding to similar temporal behavior, are the following:

- Group 1: NDVI, CIREDEDGE;
- Group 2: SAVI, IRECI;
- Group 3: PSRI;
- Group 4: VII.

### 3.3. Change Detection Maps on Selected Indices

The inter-annual change detection maps (defined by Equation (1)) are provided for the selected indices and the two seasons with the highest percentage of vegetation cover (winter, spring) (Section 3.1). The reference year is 2016. The index maps of the years 2017, 2018, 2019 and 2020 are compared with those of 2016. The statistics (median, Q1, Q3, mean, standard deviation) of the change detection maps are calculated by zone.

**Figure 15:**
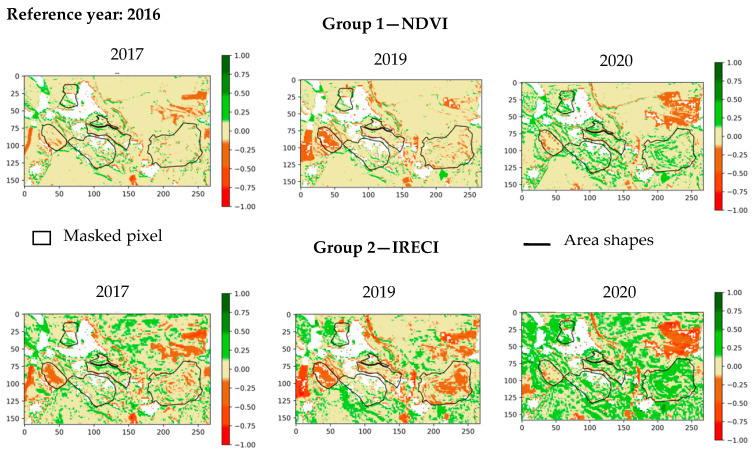
NDVI and IRECI (Inverted Red-Edge Chlorophyll Index) index change detection maps for Table 2017. 2019, 2020 compared to the year 2016. Maps of 2018 are not provided because the changes are not significant in comparison to the other years.

**Figure A3:**
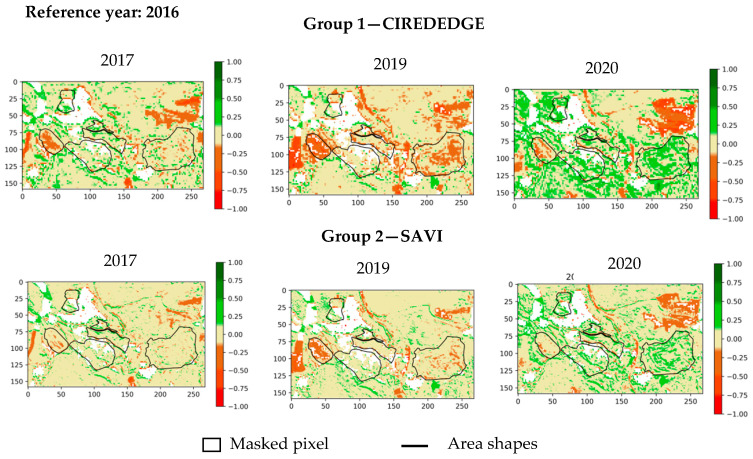
SAVI and CIREDEDGE index change detection maps for the spring of the years 2017, 2019, 2020 compared to the year 2016. Maps of 2018 are not provided because the changes are not significant in comparison to the other years. CIREDEDGE provides a better visibility of changes than NDVI. Changes are more apparent in the IRECI detection map than in the SAVI map.

**Figure A4:**
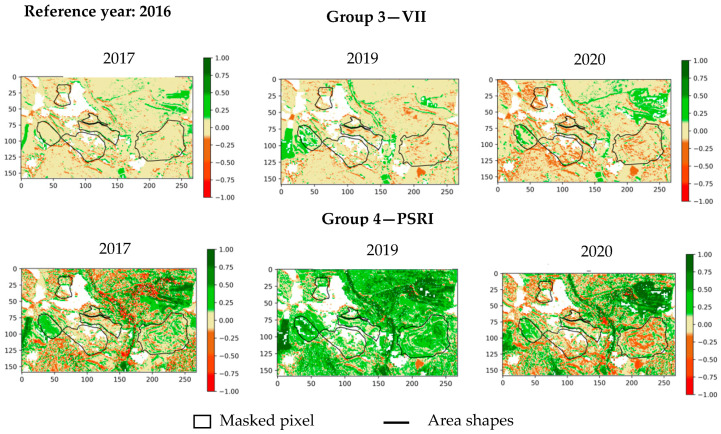
VII and PSRI index change detection maps for the spring of the years 2017, 2019, 2020 compared to the year 2016. Maps of 2018 are not provided because changes are not significant in comparison to the other years. The changes are more difficult to see in the index VII maps than in the other index maps. PSRI highlights changes by exploiting the full range of colors.

Figure 15 shows the change detection maps for NDVI and IRECI indices. CIREDEDGE provides a better visibility for changes than NDVI does (Figure 15, Figure A3). Changes are more apparent in the IRECI detection map than in the SAVI map (Figure 15, Figure A3). PSRI highlights changes from one year to another (Figure A4). The changes are more difficult to see on the index VII maps (Figure A4). In spring, the vegetation cover remains stable over the years (Table 3), but significant variations for some indices depending on the area are observed. For examples, zones 4 and 6 have a significant diminution of IRECI values between 2016 and 2019, and all areas (except zone 4) present significant increase in the IRECI between 2016 and 2020.

**Figure 16:**
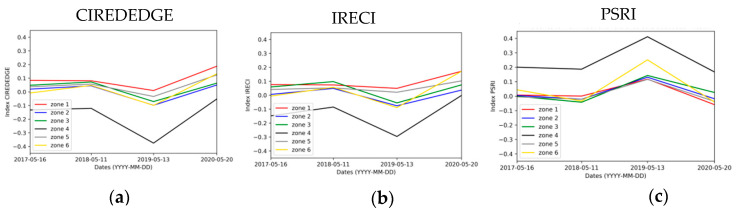
Median values of spring change detection maps on each zone for: (**a**) CIREDEDGE; (**b**) IRECI; (**c**) PSRI.

**Figure 17:**
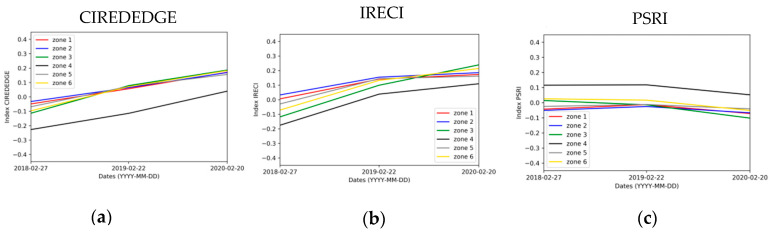
Median values of winter change detection maps one each zone for: (**a**) CIREDEDGE; (**b**) IRECI; (**c**) PSRI.

Statistics from the change detection maps are evaluated in spring and winter by zone. Figure 16 and Figure 17 illustrate the mean for the three indices CIREDEDGE, IRECI and PSRI.

For every zone (except zone 4), the NDVI, CIREDEDGE, IRECI and SAVI values remain stable or even increase significantly from spring to spring between consecutive years until 2018 (positive values < 0.1), decrease between spring 2018 and 2019 and increase until spring 2020 (deviation lower than 0.18). These variations are sensibly higher for zone 6. For zone 4, the same trend is observed but the deviation levels and temporal variations are higher. This point is confirmed in the field: this zone is undergoing regular changes. For zones 1 and 5 (phyto-stabilized), the indices present comparable temporal evolution and the evolution of the indices in phyto-stabilized zone 2 is the same as for the zone 3 with natural vegetation.

The PSRI values are roughly equal from spring 2016 to spring 2018. For zone 6, a slight decrease in index values is observed over the years until 2018. For zone 4, there is a gap between 2016 and 2017 and then stabilization until 2018. Between 2018 and 2019, PSRI values increase for every zone, which may reflect an increase in senescent vegetation during the spring season. Between 2019 and 2020, PSRI decreases and the median level values between spring 2018 and spring 2020 are comparable. This increase between 2018 and 2019 and decrease between 2019 and 2020 are twice as high for zones 4 and 6 with natural vegetation as well as for the other zones. This can be linked to the difference in terms of the dominant species in these areas. These statistics illustrate the abrupt change at the beginning of 2019, linked to the heavy rainfalls at the end of 2018.

The winter 2016 image is not available due to the presence of clouds. Only the comparison between winter 2017, winter 2019 and winter 2020 can then be performed (Figure 16).

**Figure 18:**
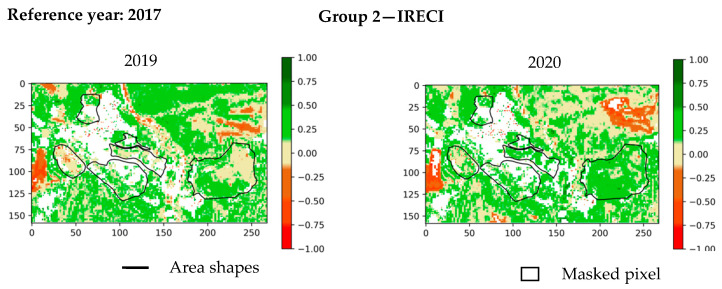
IRECI index change detection maps for the winter of the years 2019 and 2020 compared to the year 2017.

For every area, there is either a decrease in the NDVI, CIREDEDGE, IRECI, SAVI index values or these values are stable between 2017 and 2018. Globally, the median values of NDVI, CIREDEDGE, IRECI and SAVI increase from 2018 to 2020. This increase rate depends on the area and the year. Zone Z4 (natural vegetation) has lower values of NDVI, CIREDEDGE and IRECI than the other zones (on the contrary, PSRI values are higher). With regards to the IRECI index, the phyto-stabilized zones (1, 2 and 5) show particular temporal behavior: the median values increase considerably until winter 2019 (deviation around 0.18) and then stabilizes. For natural vegetation zones, IRECI median values increase between 2019 and 2020. This can be seen on the 2019 and 2020 IRECI maps (Figure 18) as values around 0 in 2019 (pale beige color) are between 0.25 and 0.5 in 2020 (lime green color) for most pixels in these zones. There is a small variation in the PSRI values between 2017 and 2020. Senescent vegetation does not increase over the years in winter.

### 3.4. Spectral and Temporal Clustering

In a first time, the time series, called annual time series, is built with all the dates of the seasons winter, spring and autumn for each year. According to the previous results (see Section 3.1), the vegetation cover is very low in summer and the corresponding dates are not retained to construct the annual time series in order to avoid distorting vegetation pattern. The change detection is applied by year. The annual time series highlights major land use classes and provides multi-year monitoring. In a second time, the time series, called seasonal time series, is built by season (including the dates of all the years for a given season) and the change detection is applied by season. The seasonal time series analysis allows highlighting phenological atypical behavior within zones.

**Figure A5:**
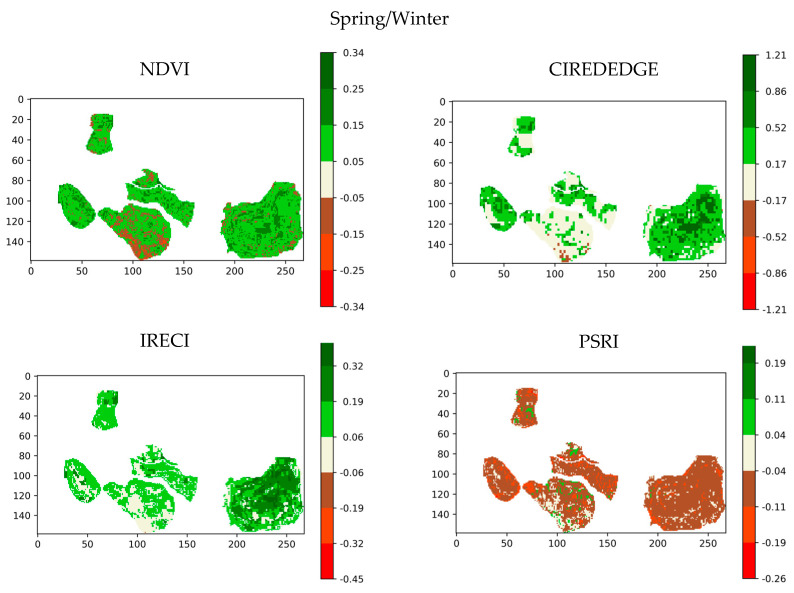
Clustering change detection maps using for seasonal time series (spring according to winter). Main differences observed: PSRI does not allow detecting change related to vegetation senescence of the south of zone 5 in spring and autumn; IRECI and CIREDEDGE highlight greater progress in spring vegetation in the center of zone 6 than NDVI; the variability of the NDVI values is higher than that of other indices.

Clustering is applied to each index of the previously selected indices and the annual time series. Change detection is then applied on the clustering maps. The results from Section 3.1 and Section 3.2 are observed: bare soils are easily recognizable, vegetation areas are globally increasing and indices follow the trends observed Figure 16, slightly increasing from 2017 to 2018, stagnating or decreasing from 2018 to 2019 and sharply increasing, especially on zone 6, until 2020. Clustering in then applied on seasonal time series in order to distinguish phenological stages. The main results previously observed are: vegetation developing in spring and decreasing in summer, except in the south of zone 5 where vegetation decreases in spring and progresses in autumn, more apparent variations in natural zones than in phyto-stabilized zones and a greater vegetation cover in zones 1 and 5 than in zone 2 in summer and autumn. Clusters of sparse vegetation vary less than the others through the seasons. These results are consistent with those already obtained in Section 3.1 (Figure 8) and Section 3.3. IRECI, CIREDEDGE, NDVI and PSRI provide similar results. The main differences are (Figure A5):

- PSRI does not allow detecting change related to the senescence of the vegetation of the south of zone 5 in spring and autumn;
- IRECI and CIREDEDGE highlight greater progress in spring vegetation in the center of zone 6 than NDVI;
- The variability of the NDVI values is higher than those of other indices.

**Figure 19:**
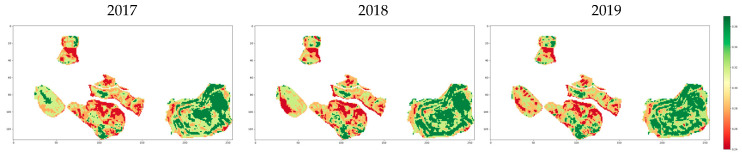
Clustering maps obtained with the combination of NDVI and PSRI for annual time series (built with dates in winter, spring and autumn of each year).

In order to take advantage of the sensibilities of spectral indices, clustering is then applied on combination of indices (Section 3.2) and the annual time series. The combination of NDVI and PSRI is retained to highlight the observed changes. Figure 19 illustrates the clustering map for each year. The size of bare soil clusters (in red) decreases while vegetation clusters (in green) are expending over the years. This progress is even more important over the entire zone 6, in the center of zone 3, in the south of zone 2 and in the south of zone 5 where areas of well-developed vegetation (dark green) spread out. Thus, both natural and phyto-stabilized vegetation seems to develop over the years. An area of bare soil is observed in the northeast of zone 1 in 2018 only. Those of zone 4 are constantly changing, a result which is consistent with those already observed (Section 3.3, Figure 15).

**Figure 20:**
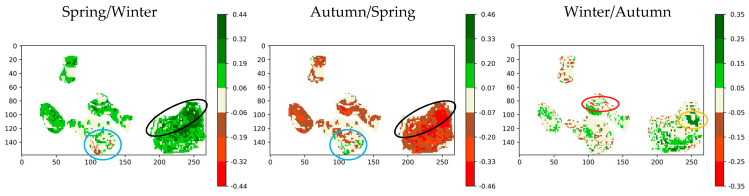
Clustering change detection maps using NDVI, CIREDEDGE, IRECI and PSRI for seasonal time series (combining all the dates of each season) (notation: season 1/season 2 means change detection map of the season 1 according to the season 2).

The spectral and temporal clustering is applied on the combination of the selected indices (CIREDEDGE, IRECI and PSRI), the reference index NDVI and the seasonal time series. The change detection maps compare season by season (Figure 20). In the south of zone 5, a phenological stage change is observed (surrounded in blue) and can be explained by the presence of grass land drying in late spring with rising temperatures and returning in autumn with rainfalls (Figure 9). In the north of zone 6 (natural zone), surrounded in black, the cluster maps always group this area in the high vegetation cluster. The index values for this area vary only slightly between spring and autumn (brown color) and increase sharply in spring (dark green color). This area seems to be composed of persistent vegetation and field observations confirm this result: the vegetation is mainly composed of Spanish brooms and pines. The vegetation in the south of zone 2 weakens strongly in winter (surrounded in red), by falling from a cluster of high vegetation to one of sparse vegetation in winter. The east of zone 5 (surrounded in orange) varies more strongly in winter and spring than the rest of the vegetation, passing from cluster of spare vegetation to high vegetation.

## 4. Discussion

The main purpose of this study is to analyze the temporal evolution of vegetation growing in an ore processing site after closure and detect the abrupt change related to one-time events (for example related to meteorological conditions) and the longer-term change related to vegetation response to stress associated to the environmental conditions of development. The vegetation is divided into two classes in this study: vegetation introduced for phyto-stabilization and natural vegetation. For that purpose, the data of Sentinel-2 satellites, several spectral indices and change detection methods are used to provide an unsupervised diagnostic tool. The time series exploited, covering four seasons and five years, leads to large volumes of images and maps to be manipulated. The change detection tool proposed, based on multi-temporal fusion at feature level and involving the normalized differences of the index maps at two dates, allows a faster and more relevant analysis of the results. The clustering approach highlights some common behaviors across the time series and could be a good way to support change detection by emphasizing general trends and unexpected variations. The results of this investigation are carried out on the basis of the vegetation classes (phyto-stabilized and natural vegetation) by qualitative and quantitative analysis of the produced maps related to spectral index or representative of vegetation cover.

Among the 26 spectral indices tested from the literature covering different contexts (environmental monitoring, precision agriculture abiotic impact…), three indices, CIREDEDGE (based on spectral bands B5 and B7 and used for the estimation of chlorophyll) [64], IRECI (including the bands B4, B5, B6 and B7 in its formulation and specified for the estimation of chlorophyll content from Sentinel-2 data) [7] and PSRI (using the bands B2, B4 and B6 and defined for senescent vegetation, sensitive to the ratio of carotenoids, carotenes and chlorophylls) [78] are particularly suitable for detecting spatial and temporal changes over the studied area. These indices usually contain a wavelength sensitive to chlorophyll (often between 660 and 720 nm) and a reference wavelength (between 750 and 800 nm) to account for vegetation structure [18]. The common spectral bands used by these three indices are defined below with the purpose for each band: B4 (maximum chlorophyll absorption), B5 (REP—Red-Edge Position), B6 (REP) and B7 (LAI—Leaf Area Index, edge of the NIR plateau) [88]. PSRI introduces the B2 band, which is sensitive to vegetation senescing, carotenoid and browning. Many previous studies employed red-edge information in order to detect spectral changes due to the high soil metal contamination [9,18,89]: our index selection confirms the usefulness of this spectra region given an indication of the chlorophyll plant status. The signal in the Red Edge as a result of the increasing chlorophyll content is due to a broadening of the absorption feature centered at 680 nm, shifting the position of the red-edge to longer wavelengths [90]. The shift in the red-edge position towards shorter wavelengths around 680 nm is then associated with a decrease in chlorophyll content. CIREDEDGE and IRECI are related to chlorophyll contents in leaf indirectly impacted by the presence of HM in the soil [4,9,18]. A high value of such indices indicates high chlorophyll content and healthy plant status. In addition to this, CIREDEDGE and PSRI are proposed as reference indices in the study conducted by Zhang and al for detecting heavy metal stress by using Sentinel-2 data [27]. IRECI has been shown to be correlated to canopy chlorophyll content and still performing well for LAI [7]. The vegetation indices selected allow capturing modifications of vegetation traits (mainly biochemical traits and LAI) even if minor vegetation cover changes are observed. In fact, during spring the vegetation cover remains relatively stable over the year, but specific spatial variations for each zone on the index map are detected and highlighted by the temporal statistical analysis.

Identifying and predicting phenological stages provide essential information for many applications. Some phenological stages are more sensitive to biotic or abiotic impacts. The Sentinel-2 time series offers a good opportunity to analyze phonological stages and transition dates [89,91,92]. The high variability of the vegetation cover cycle is analyzed in this study to detect change. Overall and abrupt changes of the vegetation cycle concerning every area, linked to heavy rainfalls, are highlighted. Vegetation development depends on meteorological conditions such as temperatures and rain fall. In special cases (floods, heatwaves, frost…), selected dates to construct the time series could be adjusted annually based on meteorological observations. A difference in the vegetation cycle across the area is observed (vegetation cover increases in autumn from 2017), likely related to the relative abundance of the different plant species. The most appropriate seasons to monitor vegetation on this study site are defined according to vegetation cover: winter and spring.

The spatial resolution offered by S2 allows to separate and analyze single areas, even when their size is relatively small (few hectares), with a high temporal resolution. Even if temporal and spectral clustering shows a reduction in bare soil surface over the years, some permanent bare soil areas on three zones without vegetation cover whatever the year and season since 2016 are highlighted. Permanent bare soils seem to be related to limestone soils that are poor in organic matter. Further in situ investigations (spectral signature measurements of bare soil area with very marked metal signature, soil texture analysis…) have to be conducted in order to identify the major factor preventing the development of vegetation (HM concentrations, high pH, low organic matter content…) and define if this factor can be detected or quantified via optical properties. Other specific changes are identified by the tool proposed—for example, the identification of the most impacted area or, conversely, the detection of the area with vigorous vegetation (most developed trees).

Only a subtle difference has been detected between phyto-stabilized and spontaneous vegetation areas (like differences noticed by IRECI in winter). No global trend can be identified for the phyto-stabilized areas and the zones with natural vegetation. This can be explained by the difference in terms of dominant species in these areas or by the differences in management methods for the aided phyto-stabilisation (partial phyto-stabilisation, tree planting, addition of natural soil, etc.). Seasonal and annual clustering gives information on land cover (bare soil, dense or sparse vegetation) and habitats (persistent, growing cycle-dependent). To remove certain ambiguities, major species and habitats on each zone have to be identified. Moreover, control zones spatially independent from the study site with similar species, soil mineralogy and exposition could be identified and the vegetation development of these control zones will be compared to the zones affected by the ore processing activity.

Most studied vegetation indices use spectral wavelengths alongside the red-edge region, sensitive to changes in chlorophyll and other pigments. This can lead to false alarms as the changes due to other biological and physiological stressors inducing chlorophyll alterations can be confused with the changes related to metal accumulation [89]. Excessive metal accumulation is known to affect leaf internal structure, cell structure and chloroplast ultrastructure [89,93], information available in the spectral domain (800–1300 nm). The combination of the red-edge domain with the SWIR domain can reduce false alarms and improve the detection of HM alteration. To further establish a direct link between HM in the soil and the health status of vegetation, in situ investigations are necessary and measurements of spectral signature of the major species with a field spectro-radiometer complemented along with measurements of leaf biochemical parameters (pigment and water contents…) and HM concentrations in soil have to be acquired in order to define the most sensitive and useful spectral bands in the whole reflective domain (400–2500 nm) according to the context defined by the HM type and level as well as the plant species [11].

Previous studies have proven the interest of hyperspectral data (typically airborne or in-field measurements) for monitoring HM stress [9,26,94]. On the one hand, the approach, proposed to assess the potential of leaf optical properties for predicting Total Petroleum Hydrocarbon concentrations and based on the retrieval of leaf pigment using the PROSPECT model, could be applied in this context on a range of plant species over a full seasonal cycle in order to identify the most suitable conditions for detecting and predicting HM concentrations [10,19]. On the other hand, it would be interesting to process an airborne hyperspectral image acquired on the site in order to analyze the contribution of spectral and high spatial (metric) resolutions in comparison to temporal repeatability.

## 5. Conclusions

An unsupervised methodology for monitoring vegetation on an ore processing site closed and revegetated is proposed. It operates multispectral and multi-temporal Sentinel-2 images and generates change detection maps based on the exploitation of spectral indices and spectral and temporal clustering. Three indices, CIREDEDGE, IRECI and PSRI, related to the biochemical traits of vegetation are particularly suitable for detecting changes spatially and temporally across the study area.

Image time series reveal its capacity for monitoring vegetation in such a context, and this study proves that the multi-date analysis are important to emphasize difference in vegetation development and vegetation trait variation. Global and specific changes are emphasized by the exploitation of spectral index maps, vegetation masks related to vegetation cover, clustering maps and change detection maps. The major changes detected are explained by information provided by the site operator or meteorological conditions.

The results are specific to the study site owing to its history, the phyto-stabilization process and the species in development but this unsupervised diagnostic tool remains valid for other contexts such as phyto-management monitoring, the analysis of restored or polluted sites or precision agriculture.

Further study is needed in order to better understand the behavior difference between the vegetation introduced for phyto-stabilization and the natural vegetation, as well as the impact of trace metals on the vegetation health. In a first time, species or habitat mapping will be of great interest. Supervised classification methods based on machine learning algorithms could then be applied to the Sentinel-2 time series. In a second time, it seems necessary to carry out multi-temporal field measurements with a spectro-radiometer combined with measurements of biochemical traits of the major species and soil parameters (texture, HM, pH…) on contaminated and control zones in order to define the combination of wavelengths optimal to characterize the metal accumulation and/or metal stress in plants.

According to the relatively small size of the study areas, the spatial distribution of vegetation, the size of the plant patches and the major species, spatial information must be exploited through high spatial resolution imagery. Unmanned aerial vehicle (UAV) and airborne platforms taking hyperspectral camera on board could then be used over time to monitor the temporal dynamics of vegetation, map species and detect altered vegetation due to soil HM by combining high spatial, spectral and spatial resolution information.

## Figures and Tables

**Table 1:** Time series of Sentinel-2 images.

Image id Number	Acquisition Date ^1^
SENTINEL2A_20160521-105553	2016/05/21
SENTINEL2A_20160826-104023	2016/08/26
SENTINEL2A_20161015-104513	2016/10/15
SENTINEL2A_20170225-105020	2017/02/25
SENTINEL2A_20170516-105322	2017/05/16
SENTINEL2A_20170821-104208	2017/08/21
SENTINEL2A_20171013-105315-	2017/10/13
SENTINEL2A_20180227-104236	2018/02/27
SENTINEL2A_20180511-105804	2018/05/11
SENTINEL2A_20180819-105124	2018/08/19
SENTINEL2A_20181025-104115	2018/10/25
SENTINEL2A_20190222-104853	2019/02/22
SENTINEL2A_20190513-104901	2019/05/13
SENTINEL2A_20190814-105857	2019/08/14
SENTINEL2A_20191030_104901	2019/10/30
SENTINEL2A_20200220-105848	2020/02/20
SENTINEL2A_20200520-105859	2020/05/20

^1^ Date format: year/month/day.

**Table 2:** Spectral indices database.

S2 Spectral Band		B1	B2	B3	B4	B5	B6	B7	B8	B11	B12
Spectral Band Formulation ^1^		Ae.	B	G	R	RE1	RE2	RE3	NIR	SWIR	SWIR
Spectral Index	Description										
ARVI	Atmospherically Resistant Vegetation Index [62]		x		x			x			
C_1D	Maximum of first derivate reflectance at the red edge [63].				x	x	x	x			
CIREDEDGE	Red-edge Chlorophyll Index [64]					x		x			
CTR	Carter index [65]				x		x				
EMEN2	Red and green ratio [66]			x	x						
FIRSTDV_CHL	Ratio of the derivative at red-edge [67]					x	x				
GREEN_NDVI	Green NDVI [68]			x				x			
HMSSI	Heavy Metal Stress Sensitive Index [27]		x		x	x	x	x			
IRECI	Inverted Index Red-Edge Chlorophyll Index [7]				x	x	x	x			
mARI	Modified Anthocyanin Reflectance Index [69]			x		x		x			
MCARI	Modified Chlorophyll Absorption Ratio Index [70]			x	x	x					
MCARI_NEW	New MCARI [71]			x		x		x			
MCARI_OSAVI	MCARI/Optimized SAVI [72]			x	x	x		x			
MCARI_OSAVI_NEW	[72]			x		x	x	x			
MSAVI	Modified Soil Adjusted Vegetation Index [73]				x			x			
MTCI	MERIS Terrestrial Chlorophyll Index [74]				x	x	x				
NBR	Normalized Burn Ratio [75]								x		x
NDRE	Normalized Difference Red Edge [76]					x		x			
NDVI	Normalized Difference Vegetation Index [77]				x			x			
PSRI	Plant Senescence Reflectance Index [78]		x		x		x				
S2REP	Sentinel-2 red-edge position [7]				x	x	x	x			
SAVI	Soil Adjusted Vegetation Index [60]				x				x		
SIPI	Structure Insensitive Pigment Index [79]	x			x				x		
TCARI_OSAVI	[72,79]			x	x	x		x			
TCARI_OSAVI_NEW	[72]			x		x	x	x			
VII	Vegetation Inferiority Index [80]		x	x	x	x	x	x	x		

^1^ Spectral band formulation: Ae (Coastal aerosol), B (Blue), G (Green), R (Red), RE (Vegetation Red Edge), NIR (Near-InfraRed), SWIR (Short Wave InfraRed).

**Table 3:** Rate of vegetation cover increase by season between 2016 (or 2017 in winter) and 2020. The summer results are not provided owing to the low vegetation cover.

Zone	Increase in Vegetation Cover Pixels (%)		
	Spring	Autumn	Winter
Z1 *	5	30	14
Z2 *	1	23	16
Z3	1	33	12
Z4	0	31	2
Z5 *	6	26	13
Z6	0	43	3

(*) Phyto-stabilized areas.

**Figure A2:**
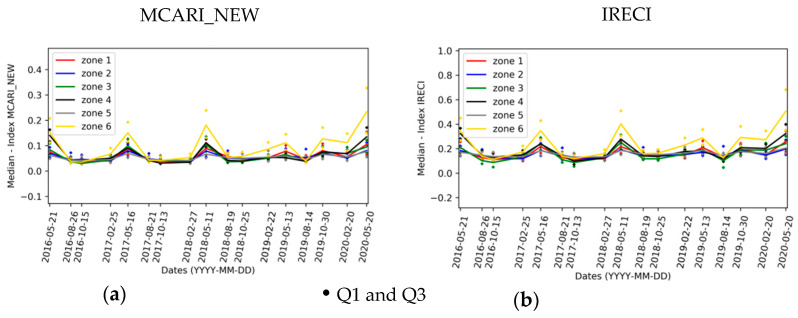
Multi-annual evolution of statistics (median, Q1, Q3) by zone: (**a**) MCARI_NEW; (**b**) IRECI. The variation range of MCARI_NEW is lower (mean values between 0.02 and 0.18) than those of NDVI. IRECI presents the temporal variability of interest for change detection.

## Appendix A

This part provides supplementary results to complete the results analysis and interpretation.
